# Supplementary material for: miR-155 harnesses Phf19 to potentiate cancer immunotherapy through epigenetic reprogramming of CD8+ T cell fate
Source: Nat Commun. 2019 May 14;10:2157. doi: 10.1038/s41467-019-09882-8 (PMC6517388; doi:10.1038/s41467-019-09882-8)
Supplement: Supplementary file 1 — Supplementary Information [file 41467_2019_9882_MOESM1_ESM.pdf]

miR-155 harnesses Phf19 to potentiate cancer immunotherapy through epigenetic reprogramming of $CD8^+$ T cell fate

Ji, Fioravanti, Zhu et al.

Supplementary Information

Supplementary Fig. 1

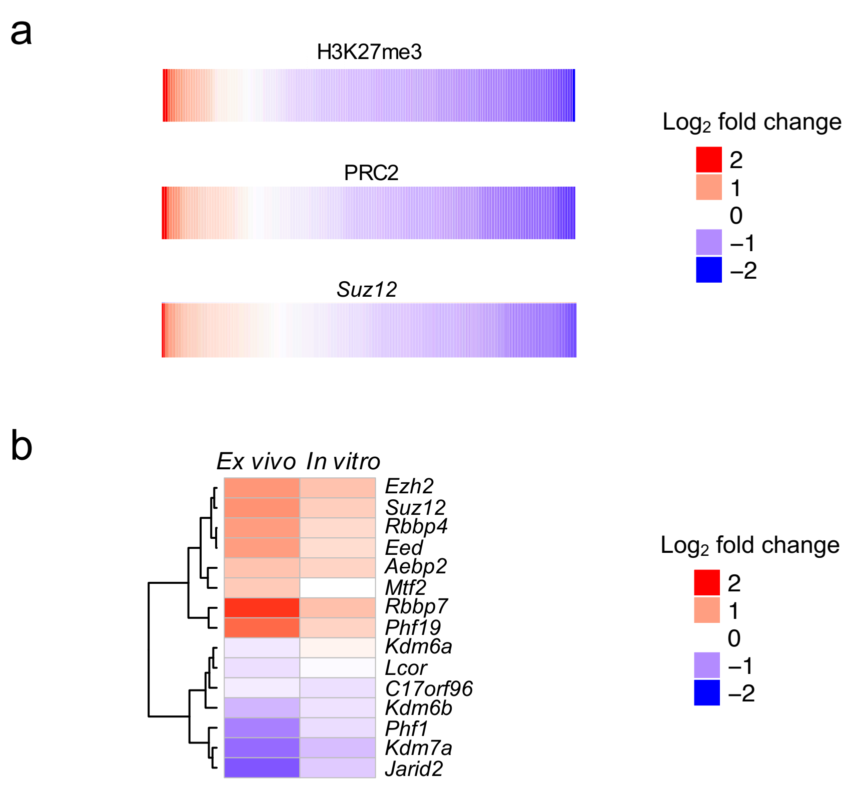

Supplementary Fig. 1. miR-155 overexpression regulates the H3K27me3 machinery. **a** Expression levels of molecules targeted by H3K27me3 (top)[20], PRC2 (middle)[21] and Suz12 (bottom)[21] by RNA-seq of pmel-1 $CD8^+$ T cells transduced with miR-155 or Ctrl-miR 5 days after *in vivo* stimulation with gp100-VV. **b** Expression levels of molecules regulating H3K27me3 deposition by RNA-seq of pmel-1 $CD8^+$ T cells as in **a** or after 5 days of *in vitro* culture. Data are presented as a Log_2 fold change relative to Ctrl-miR.

Supplementary Fig. 2

a

Biological Functions	*p*-Value	Activation z-score	# Molecules
cell cycle progression	1.35E-29	3.343	136
DNA replication	2.13E-24	3.069	55
M phase	1.4E-21	2.123	47
repair of DNA	1.57E-19	2.895	49
checkpoint control	7.97E-19	2.224	31
metabolism of DNA	8.48E-19	3.536	64
proliferation of cells	3.86E-15	5.763	270
cell death	5.45E-12	-2.606	244

b

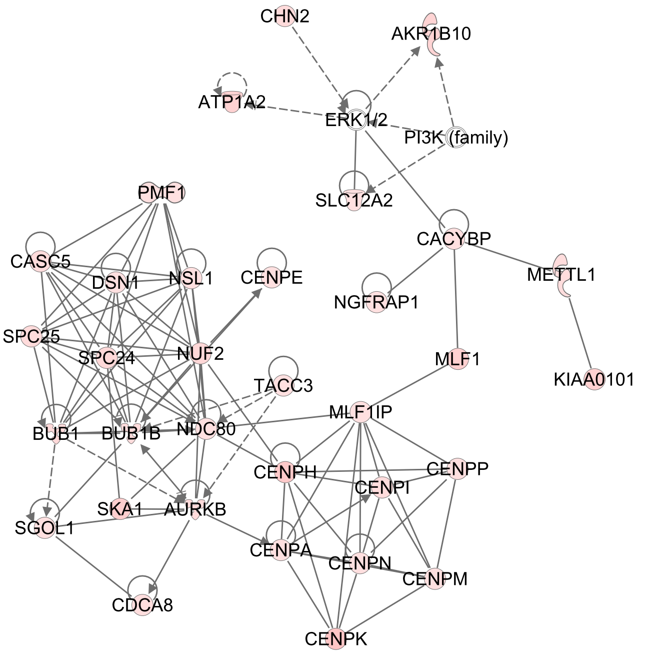

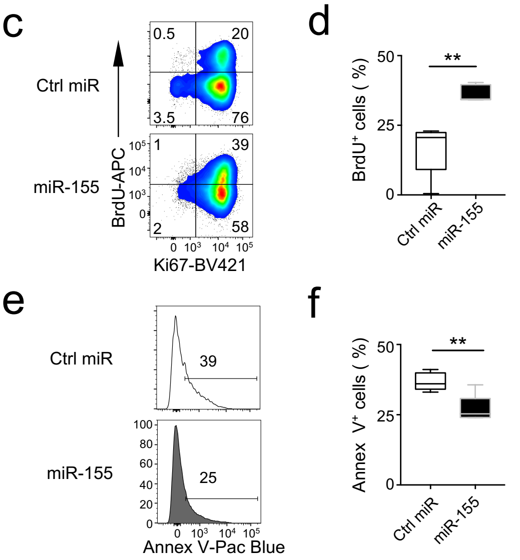

Supplementary Fig. 2. miR-155 enhances CD8⁺ T cell proliferation and survival**. a** Top-scoring biological functions differentially regulated between miR-155 and Ctrl-miR pmel-1 CD8⁺ T cells 5 days after *in vivo* stimulation with gp100-VV identified with the IPA software. **b** Cell cycle, cellular assembly and organization, DNA replication, recombination and repair network. Upregulated genes are displayed in red. Solid and dashed lines between genes represent known direct and indirect gene interactions, respectively. The shapes of the nodes reflect the functional class of each gene product. **c**–**d** Flow cytometry (**c**) and percentage (**d**) of BrdU incorporation into pmel-1 cells 5 days after adoptive transfer of 3 X 10^5 miR-155 and Ctrl-miR pmel-1 CD8⁺ T cells into wild-type mice infected with gp100-VV. Numbers (**c**) indicate the percentages of cells in each quadrant after gating on live CD8⁺GFP⁺ T cells. **e**–**f** Flow cytometry (**e**) and percentage (**f**) of Annexin V⁺ pmel-1 cells 6 days after adoptive transfer as in **c**–**d**. Numbers (**e**) indicate the percentage of Annexin V⁺ cells after gating on CD8⁺GFP⁺ T cells. ** = *P* < 0.01 (unpaired two-tailed Student's *t*-test).

Supplementary Fig. 3

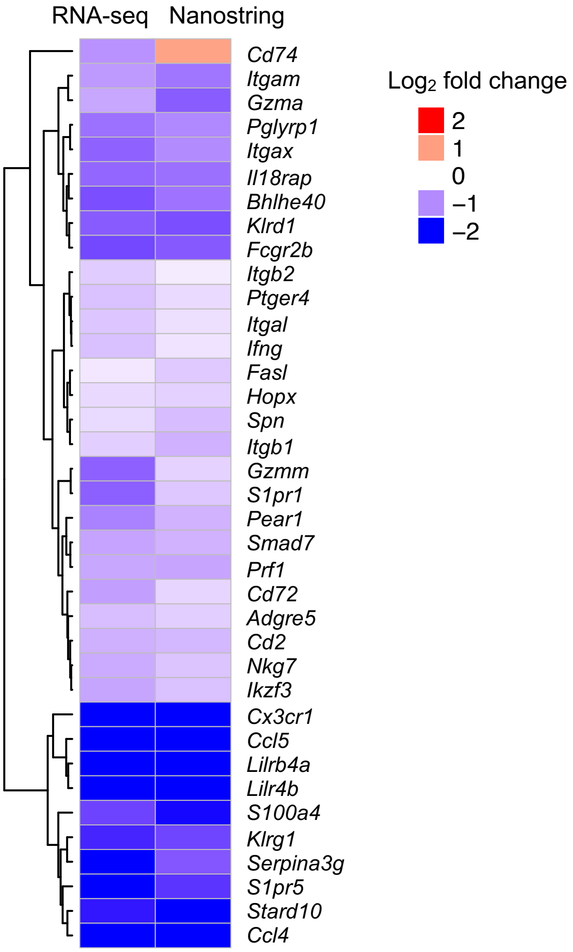

Supplementary Fig. 3. miR-155 overexpression restrains CD8^{+} T cell terminal differentiation. Expression levels of inhibitory receptors, cytotoxic molecules, and chemokines known to be highly expressed in terminally differentiated CD8^{+} T cells in miR-155 and Ctrl cells, evaluated by RNA-seq and Nanostring 5 days after transfer of pmel-1 CD8^{+} T cells transduced with miR-155 or Ctrl-miR into mice infected with gp100-VV. RNA-seq data were obtained from the RNA of 5 pooled mice/group and Nanostring was done in duplicate on RNA samples from 3 pooled animals/group (miR-155 vs Ctrl-miR). Data are presented as a Log_2 fold change relative to Ctrl or Ctrl-miR.

Supplementary Fig. 4

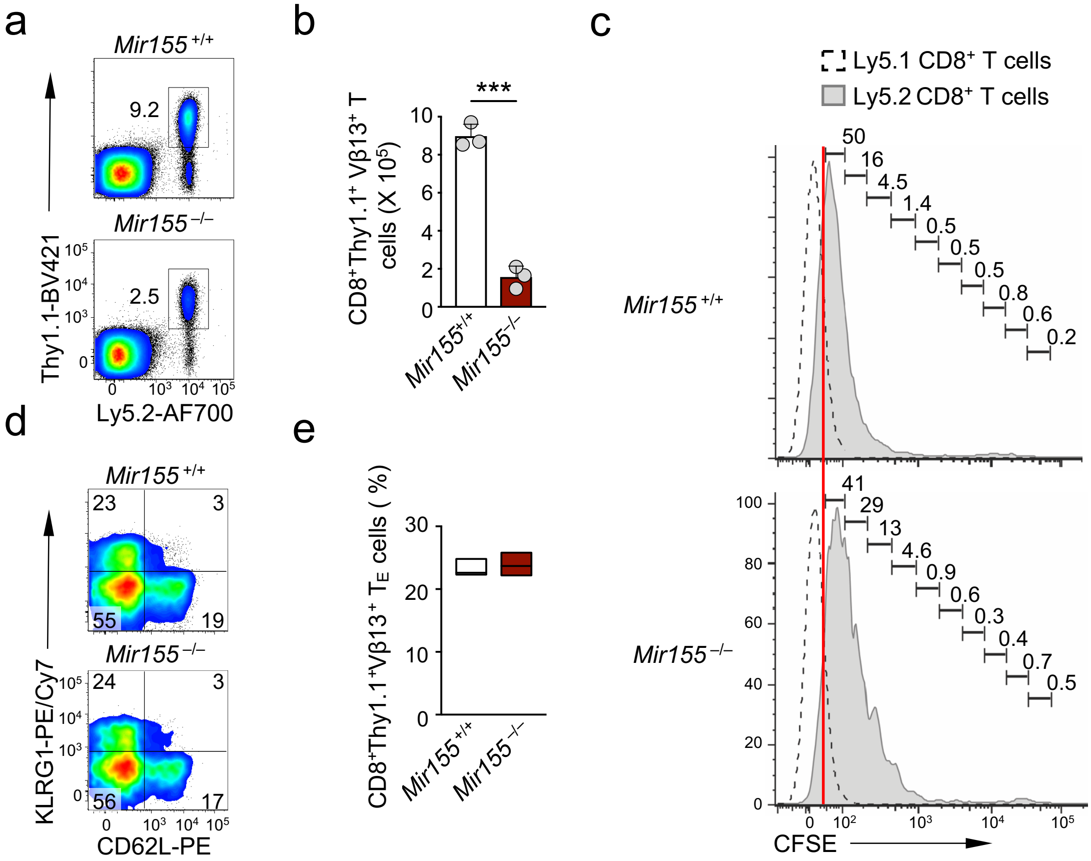

Supplementary Fig. 4. miR-155 restricts CD8⁺ T cell terminal differentiation. **a** Flow cytometry of splenic CD8⁺ T cells 4 days after transfer of 1.5 x 10^5 pmel-1 TCR transduced CFSE-labeled *Mir155*$^{+/+}$ and *Mir155*$^{-/-}$ CD8⁺ T cells into Ly5.1 mice in conjunction with gp100-VV. Numbers indicate the percentage of cells after gating on live CD8⁺T cells. **b** Number of live Thy1.1⁺ Vβ13⁺ T cells in *Mir155* sufficient or deficient CD8⁺ T cells after transfer as in **a**. **c** CFSE dilution of *Mir155* sufficient or deficient CD8⁺ T cells after transfer as described in **a**. Numbers indicate the percentage of cells per each generation. **d** Flow cytometry of CD8⁺Thy1.1⁺Vβ13⁺ T cells after transfer as in **a**. Numbers indicate the percentage of cells in each quadrant after gating on live CD8⁺T cells. **e** Percentage of pmel-1 CD8⁺Thy1.1⁺Vβ13⁺ T_E cells after transfer as in **a**. Data are presented as box plots extending as a range. Bands inside the boxes represent median values of three mice. Data are presented as the mean of three mice in two independent experiments. *** = $P < 0.005$ (unpaired two-tailed Student's t-test).

Supplementary Fig. 5

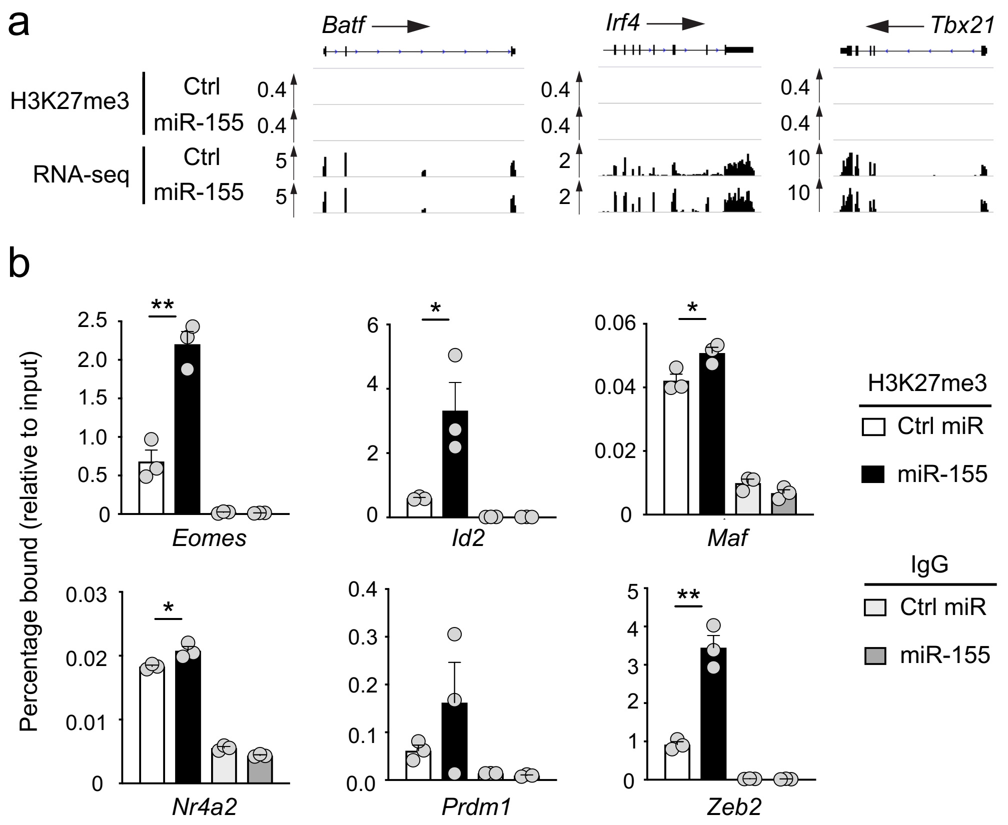

Supplementary Fig. 5. miR-155 overexpressing cells demonstrate increased H3K27me3 deposition at the transcription start site of TFs known to promote terminal differentiation. **a** ChIP-seq of Non T_E KLRG1⁻ pmel-1 CD8⁺ T cells transduced with miR-155 or Ctrl-miR and cultured *in vitro* for 5 days. RNA-seq of KLRG1⁻CD62L⁻ cells sorted 5 days after transfer of 3 x 10^5 miR-155 or Ctrl-miR-overexpressing pmel-1 cells into wild-type mice in conjunction with gp100-VV. RNA-seq data were obtained from triplicated groups of three individual mice. **b** ChIP-qPCR of miR-155 or Ctrl-miR-overexpressing pmel-1 cells with primers specific to the TSS of selected TFs. ChIP enrichments are presented as the percentage of protein bound, normalized to input. Bars represent the mean ± s.e.m. of technical triplicates. Data are representative of two independent experiments. * = $P < 0.05$; ** = $P < 0.01$ (unpaired two-tailed Student's *t*-test).

Supplementary Fig. 6

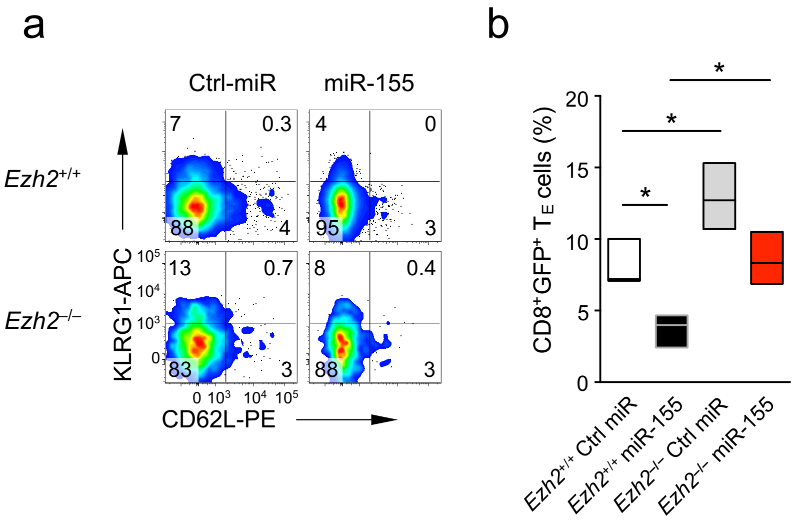

Supplementary Fig. 6. Ezh2 is essential for restricting CD8+ T cell exhaustion within the tumor. **a** Flow cytometry of intratumoral CD8+GFP+ T cells 5 days after transfer of 3 x 10^5 pmel-1 *Ezh2*+/+ and *Ezh2*–/– cells transduced with miR-155 or Ctrl-miR into tumor-bearing wild-type mice in conjunction with gp100-VV. Numbers indicate the percentage of cells after gating on live CD8+GFP+ T cells. **b** Percentage of live pmel-1 CD8+GFP+KLRG1+ T_E cells in the tumor 5 days after transfer as described in **a**. Data are presented as box plots extending as a range. Bands inside the boxes represent median values of three mice. Data are presented as the mean of three to four mice in two independent experiments.
* = $P < 0.05$ (unpaired two-tailed Student's *t*-test).

Supplementary Fig. 7

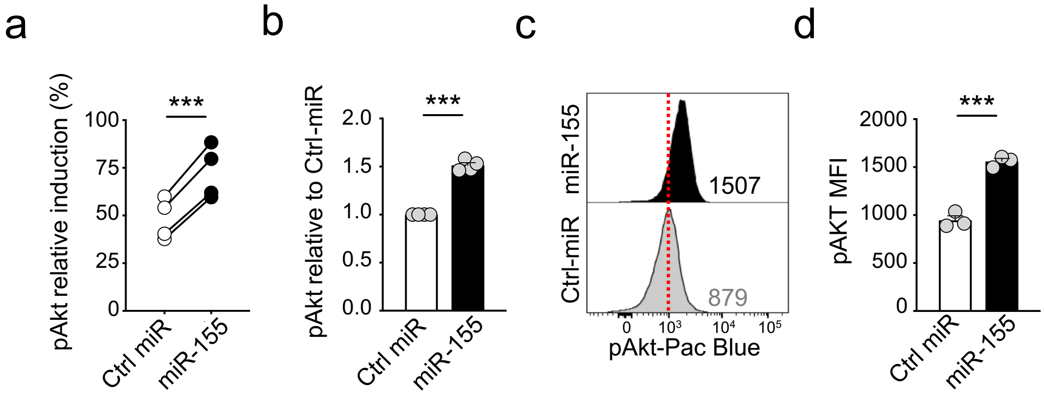

Supplementary Fig. 7. miR-155 enhances pAkt signaling **a.** pAkt induction in Ctrl-miR or mir-155 overexpressing cells assessed by immunoblot in *in vitro* activated cells. Data are relative to Gapdh. Representation of four independent experiments. **b.** Fold increase of pAkt levels in miR-155 overexpressing cells relative to Ctrl-miR, shown in **a**. **c.** Histograms of pAkt levels of *in vitro* activated Ctrl-miR or miR-155 overexpressing cells assessed by intracellular flow cytometry. Numbers indicate the mean fluorescence intensity (MFI) after gating on live $CD8^{+}GFP^{+}$ T cells. **d.** Mean fluorescence intensity (MFI) of pAkt in *in vitro* activated Ctrl-miR or miR-155 overexpressing cells. *** = $P < 0.005$ (paired two-tailed Student's t-test) (**a** and **b**) *** = $P < 0.005$ (unpaired two-tailed Student's t-test) (**d**)

Supplementary Fig. 8

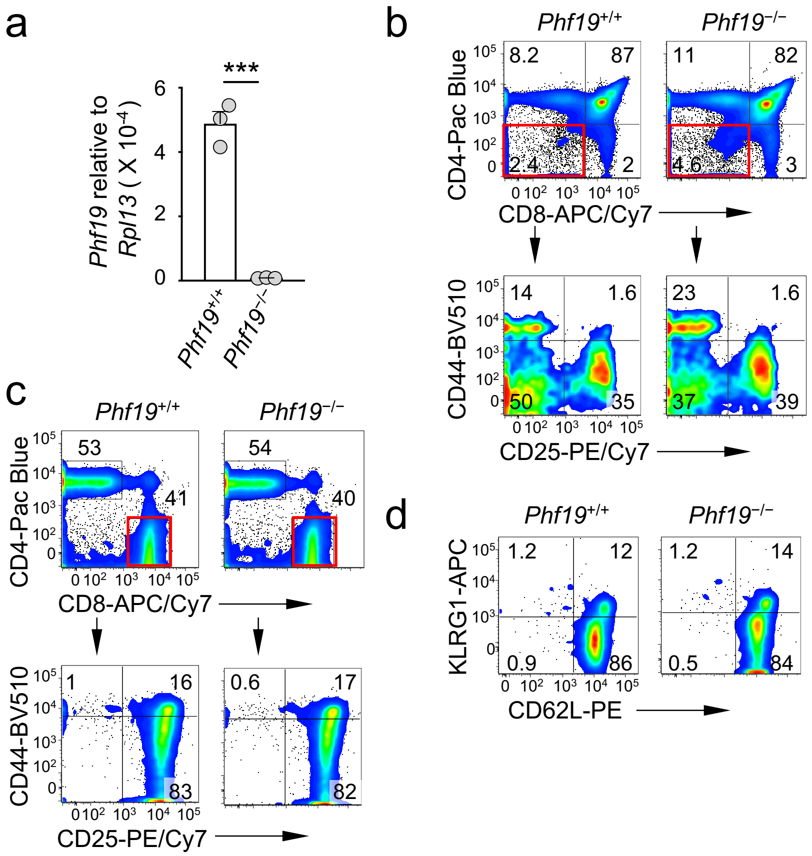

Supplementary Fig. 8. *Phf19* deficient animals exhibit normal T cell development and homeostasis. **a** Quantitative RT-PCR of *Phf19* levels in *Phf19*$^{+/+}$ or *Phf19*$^{-/-}$ CD8$^+$ T cells. Bars (mean ± s.e.m. of technical triplicates) are relative to *Rpl13* mRNA. **b** Flow cytometry of thymocytes from *Phf19*$^{+/+}$ or *Phf19*$^{-/-}$ mice. Numbers indicate percentage after gating on CD3$^+$ cells (top), CD3$^+$CD4$^-$CD8$^-$cells (bottom). **c** Flow cytometry of splenocytes from *Phf19*$^{+/+}$ or *Phf19*$^{-/-}$ mice. Numbers indicate percentage after gating on CD3$^+$ cells (top), CD3$^+$CD8$^+$ cells (bottom). **d** Flow cytometry analysis of splenocytes from pmel-1 *Phf19*$^{+/+}$ or *Phf19*$^{-/-}$ mice. Numbers indicate percentage after gating on CD8$^+$ cells. Data are presented as the mean of three to four individual mice in two independent experiments. *** = $P < 0.005$ (unpaired two-tailed Student's *t*-test).

Supplementary Fig. 9

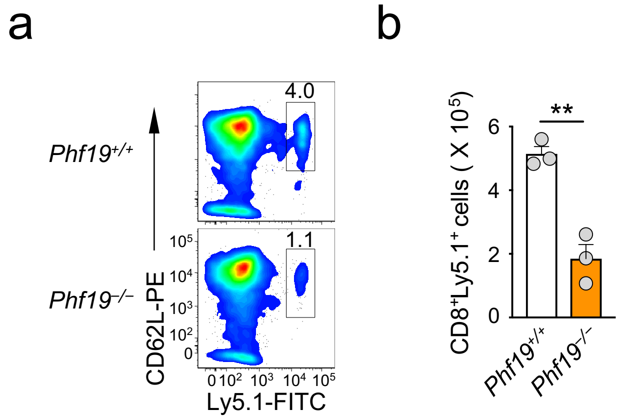

Supplementary Fig. 9. Phf19 regulates CD8$^+$ T cell memory formation. **a**,**b** Flow cytometry (**a**) and number (**b**) of splenic CD8$^+$Ly5.1$^+$ T cells after 30 days of adoptive transfer of 10^5 *Phf19*$^{+/+}$ and *Phf19*$^{-/-}$ naïve pmel-1 CD8$^+$ T cells into wild-type mice in conjunction with gp100-VV. Numbers indicate percentage after gating on live CD8$^+$Ly5.1$^+$ T cells. Bars represent the mean ± s.e.m. of three mice. Data are presented as the mean of three to four mice in two independent experiments. ** = $P < 0.01$ (unpaired two-tailed Student's *t*-test).

Supplementary Fig. 10

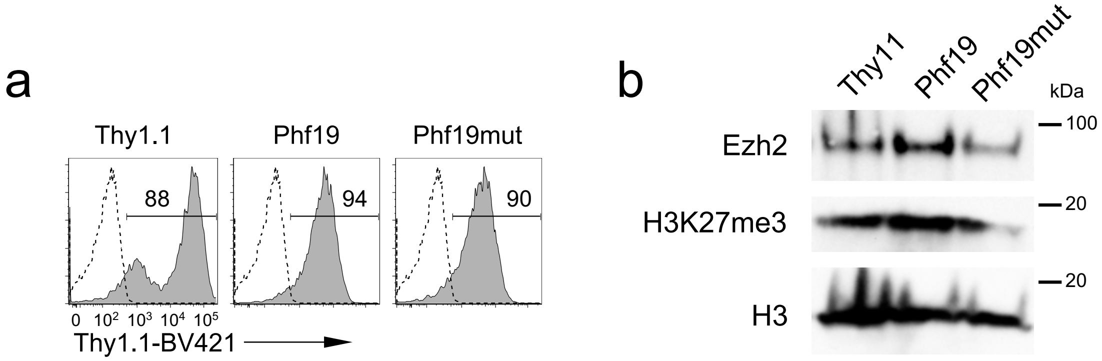

Supplementary Fig. 10. Tryptophan 41 (W41) and tyrosine 47 (Y47) in the aromatic cage of Phf19 Tudor domain mediate chromatin binding. **a** Flow cytometry of $CD8^+$ T cells overexpressing Phf19, Phf19mut, or Thy1.1. Numbers indicate percentage of $Thy1.1^+$ cells after gating on $CD8^+$ T cells. **b** immunoblot of Ezh2, H3K27me3, and H3 levels in chromatin fraction of $CD8^+$ T cells transduced with Phf19, Phf19mut or Thy1.1.

Supplementary Fig. 11

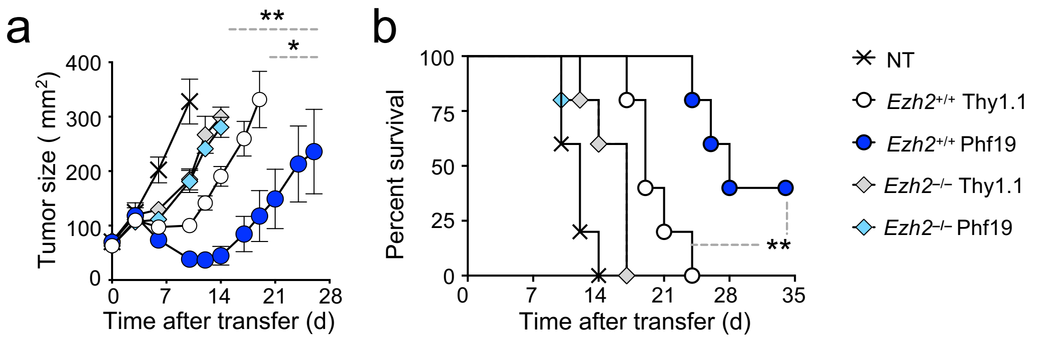

Supplementary Fig. 11. Ezh2 is required for the enhanced CD8$^+$ T cell antitumor immunity conferred by Phf19. **a**, **b** Tumor size (mean ± s.e.m.) (**a**) and survival (**b**) of B16 tumor-bearing mice after adoptive transfer of 4 x 10^5 pmel-1 *Ezh2*$^{+/+}$ or *Ezh2*$^{-/-}$ CD8$^+$ T cells transduced with Phf19Thy1.1 or Thy1.1 in conjunction with gp100-VV and IL-2 (*n*= 5 mice/group). Data are representative of two independent experiments. NT, no treatment * = P < 0.05; ** = P < 0.01 (unpaired two-tailed Student's t-test) (**a**) ** = P < 0.01 [a Log-rank (Mantel-Cox) Test] (**b**).

Supplementary Fig. 12

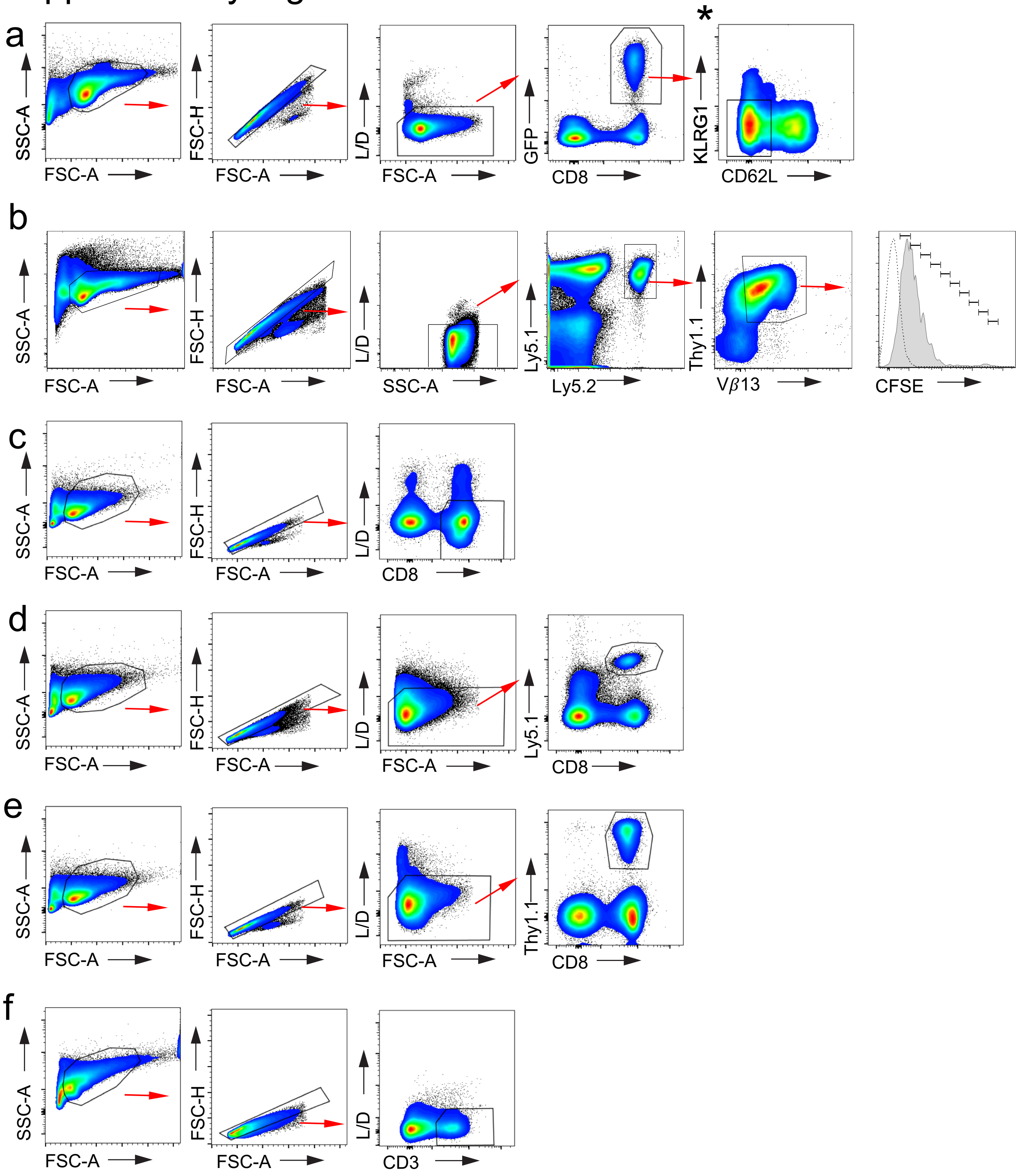

Supplementary Fig. 12. Gating strategies used for flow cytometry analysis and sorting in **a** Fig. 1c-e, 2a-d, 3d-g, 6e-g, and Suppl. Fig. 2c-f, 6a,b 7c,d **a*** gating was followed for sorting of non-skewed populations in Fig 1h **b** Fig. 1f,g, and Suppl. Fig. 4b-e **c** Fig. 4e, and Suppl. Fig. 4a, 8d **d** Fig 5b-d, and Suppl. Fig. 9a,b **e** Fig 7d-h (for persistence in Fig 7c,d Thy1.1^{+} in total live cells is shown) and Suppl. Fig. 10a. **f** Suppl. Fig. 8b,c.

Supplementary Table 1. Primer sequences

Primers	Sequence (5'-3')
Eed F	TGGCCTACAATCTGAACTCCC
Eed R	GACCACACTCTGCAAAGAAGT
Ezh2 F	AGTGACTTGGATTTTCCAGCAC
Ezh2 R	AATTCTGTTGTAAGGGCGACC
Suz12 F	AACTCGAAATCTTATCGCACCAA
Suz12 R	TGCAAATGTGCAGACAAGCTAT
Jarid2 F	ATGACAGCGATGGGATCC
Jarid2 R	GCTGCCTTTTCTGTGCATTC
Phf19 F	TGACAGAGGGACAGTTCGTG
Phf19 R	GATCTCGTTCATAGGCCCTGA
Rpl13 F	CGAGGCATGCTGCCCCACAA
Rpl13 R	AGCAGGGACCACCATCCGCT
miR-155	mmu-miR-155-5p (002571, Life Technologies)
U6	U6 snRNA (001973, Life Technologies)
Phf19shRNA F	CCGGCCTAGCCAGTATATTCGACTTCTCGAGAAGTCGAATATACTGGCTAGGTTTTTG
Phf19shRNA R	AATTCAAAAACCTAGCCAGTATATTCGACTTCTCGAGAAGTCGAATATACTGGCTAGG
Ship1 gRNA F	CCGGAGCACCTGAAAGCCATCCGTTTTAGAGCTAG
Ship1 gRNA R	GATCCTAGCTCTAAAACGGATGGCTTTCAGGTGCTCC

ChIP-qPCR Primers	Sequence (5'-3')
Eomes F	GAGCTTGCTCTAGGGGTAGG (Chromosome 9: 118,476,227-118,476,247)
Eomes R	ACAGCCAGAAGTAAGGTCCC (Chromosome 9: 118,476,388-118,476,408)
Id2 F	CGCCACAATTCCGACCTTAG (Chromosome 12: 25,100,336-25,100,356)
Id2 R	AAATATTTGCGGCGCTCCAT (Chromosome 12: 25,100,545-25,100,565)
Prdm1 F	TTGGGGCACAGATACCATGT (Chromosome 10: 44,460,649-44,460,669)
Prdm1 R	TCCTCCCTAGACTCAAGCCT (Chromosome 10: 44,460,841-44,460,861)
Maf F	CTGCAGACATTTTGAGGCGT (Chromosome 8: 118,236,772-118,236,792)
Maf R	TCTAACTGAGCCGGTGTTGT (Chromosome 8: 118,236,930-118,236,950)
Nr4a2 F	TGGTTGTTCTAGGGCGTGAT (Chromosome 2: 56,944,999-56,945,019)
Nr4a2 R	TACCCGGCCAAACTCTCAAT (Chromosome 2: 56,944,999-56,945,019)
Zeb2 F	TGAAATTCCACCTCCCTCCC (Chromosome 2: 56,945,135-56,945,155)
Zeb2 R	TCCCTTTAACTTTCGCCCCT (Chromosome 2: 45,113,439-45,113,459)

Supplementary Table 2. Flow Cytometry and immunoblot antibodies

Flow cytometry antibodies

Antibody	Clone	Source	Dilution	Catalogue
anti-CD8α -APC/Cy7	53-6.7	Biolegend	1/200	100714
anti-KLRG1-APC/ or PE/Cy7	2F1	Biolegend	1/200	138412/138416
anti-CD4-Pac Blue	GK1.5	Biolegend	1/200	100428
anti-Ly5.1-FITC or APC/Cy7	A20	Biolegend	1/200	110706/110716
anti-BrdU-APC	Bu20a	Biolegend	1/100	339808
anti-Ki67-BV421	16A8	Biolegend	1/100	652411
anti-Ly5.2-AF700	104	Biolegend	1/200	109822
anti-Annexin V-Pac Blue		Biolegend	1/100	640918
anti-IL-2-PE	JES6-5H4	Biolegend	1/200	503808
anti-CD3-APC	145-2C11	Biolegend	1/200	100312
anti-CD62L-PE	MEL-14	BD Bioscience	1/200	553151
anti-CD44-BV510	1M7	BD Bioscience	1/200	563114
anti-IFNγ-AF700	XMG1.2	BD Bioscience	1/200	557998
anti-TNF-APC	MP6-XT22	BD Bioscience	1/100	554420
anti-CD8α-PerCP/Cy5.5	53-6.7	BD Bioscience	1/200	551162
anti-Vβ13 TCR-APC	MR12-3	BD Bioscience	1/100	561542
anti-CD25-PE/Cy7	PC61.5	eBioscience	1/200	25-0251-82
anti-Thy1.1-eFluor450	HIS51	eBioscience	1/200	48-0900-82
anti-pAkt1 (Ser473)-eFluor450	SDRNR	eBioscience	1/100	48-9715-42

Immunoblot antibodies

Antibody	Clone	Source	Dilution	Catalogue
anti-pAkt	193H12	Cell Signaling	1/1000	4058S
anti-Ship1	D1163	Cell Signaling	1/1000	2728S
anti-Ezh2	D2C9	Cell Signaling	1/1000	5246S
anti-Suz12	D39F6	Cell Signaling	1/1000	3737S
HRP-anti–mouse IgG	Polyclonal	Cell Signaling	1/3000	7076S
HRP-anti–rabbit IgG	Polyclonal	Cell Signaling	1/3000	7074S
anti-Jarid2	Polyclonal	Abcam	1/1000	Ab48137
anti-H3	Polyclonal	Abcam	1/10000	Ab1791
anti-IgG	Polyclonal	Abcam	1/3000	Ab46540
anti-H3K27me3	Polyclonal	Millipore	1/1000	07-449
anti-Gapdh	6C5	Millipore	1/3000	Mab374
anti-V5	R960-25	Invitrogen	1/1000	R960-25
anti-Actin	C4	Santa Cruz Biotechnology	1/1000	SC-47778
