# Supplementary material for: miR-155 harnesses Phf19 to potentiate cancer immunotherapy through epigenetic reprogramming of CD8+ T cell fate
Source: Nat Commun. 2019 May 14;10:2157. doi: 10.1038/s41467-019-09882-8 (PMC6517388; doi:10.1038/s41467-019-09882-8)
Supplement: Supplementary file 3 — Reporting Summary [file 41467_2019_9882_MOESM3_ESM.pdf]

nature research

Corresponding author(s): Yun Ji, Luca Gattinoni

Last updated by author(s): April 1, 2019

Reporting Summary

Nature Research wishes to improve the reproducibility of the work that we publish. This form provides structure for consistency and transparency in reporting. For further information on Nature Research policies, see Authors & Referees and the Editorial Policy Checklist.

Statistics

For all statistical analyses, confirm that the following items are present in the figure legend, table legend, main text, or Methods section.

n/a	Confirmed	
☐	☒	The exact sample size (*n*) for each experimental group/condition, given as a discrete number and unit of measurement
☐	☒	A statement on whether measurements were taken from distinct samples or whether the same sample was measured repeatedly
☐	☒	The statistical test(s) used AND whether they are one- or two-sided<br>*Only common tests should be described solely by name; describe more complex techniques in the Methods section.*
☒	☐	A description of all covariates tested
☒	☐	A description of any assumptions or corrections, such as tests of normality and adjustment for multiple comparisons
☐	☒	A full description of the statistical parameters including central tendency (e.g. means) or other basic estimates (e.g. regression coefficient) AND variation (e.g. standard deviation) or associated estimates of uncertainty (e.g. confidence intervals)
☒	☐	For null hypothesis testing, the test statistic (e.g. *F*, *t*, *r*) with confidence intervals, effect sizes, degrees of freedom and *P* value noted<br>*Give P values as exact values whenever suitable.*
☒	☐	For Bayesian analysis, information on the choice of priors and Markov chain Monte Carlo settings
☒	☐	For hierarchical and complex designs, identification of the appropriate level for tests and full reporting of outcomes
☒	☐	Estimates of effect sizes (e.g. Cohen's *d*, Pearson's *r*), indicating how they were calculated

Our web collection on statistics for biologists contains articles on many of the points above.

Software and code

Policy information about availability of computer code

Data collection	BD FACSDivaTM 8.0, BioRADXF Manager 3.0, Illumina Casava suite v1.7, nSolver 3.1.0.1
Data analysis	Flowjo v9.9.4 and v10.5, GraphPad Prism 7 and 8, Microsoft Excel, R package,TopHat 2.1.1,Bowtie 2.2.8, Sicer 1.1, edgeR, DSEq2, GSEA 3.0, Ingenuity Pathway Analysis 2.4.10, Picard v1.139, IGV 2.3.92, Samtools v1.2,nCounter

For manuscripts utilizing custom algorithms or software that are central to the research but not yet described in published literature, software must be made available to editors/reviewers. We strongly encourage code deposition in a community repository (e.g. GitHub). See the Nature Research guidelines for submitting code & software for further information.

Data

Policy information about availability of data

All manuscripts must include a data availability statement. This statement should provide the following information, where applicable:

- Accession codes, unique identifiers, or web links for publicly available datasets
- A list of figures that have associated raw data
- A description of any restrictions on data availability

Provide your data availability statement here.

Field-specific reporting

Please select the one below that is the best fit for your research. If you are not sure, read the appropriate sections before making your selection.

☒ Life sciences ☐ Behavioural & social sciences ☐ Ecological, evolutionary & environmental sciences

Life sciences study design

All studies must disclose on these points even when the disclosure is negative.

Sample size	For tumor experiments we employed 5-7 animal/group. For T cell kinetic and functional studies we employed 2-4 animals/group at each time point. These sample sizes allow for statistically valid comparisons based on previous studies conducted by this laboratory. RNA-seq were done in triplicate samples of three animals (KLRG1negCD62Lneg Phf19 WT versus KLRG1negCD62Lneg Phf19KO; KLRG1negCD62Lneg miR-155 vs KLRG1negCD62Lneg Ctrl miR) or on a single sample from 5 pooled animals/group (bulk miR-155 vs bulk Ctrl). Chip-seq was performed on a single sample. Nanostring was done in duplicate on RNA samples from 3 pooled animals/group (miR-155 vs Ctrl miR).
Data exclusions	1 RNA-seq sample in the Phf19 wt condition was excluded as it behaves as an outlier compared to the other two replicates.
Replication	All data except sequencing results were reliably reproduced in at least two independent experiments. Key results from RNA-seq or ChIP-seq were independently validated in qPCR or ChIP-PCR experiments.
Randomization	For all animal experiments, mice were randomly assigned to different treatment and control groups.
Blinding	Tumor size were blindly measured. All other data were acquired and analyzed in a non-blind fashion because did not involve subjective measurements.

Reporting for specific materials, systems and methods

We require information from authors about some types of materials, experimental systems and methods used in many studies. Here, indicate whether each material, system or method listed is relevant to your study. If you are not sure if a list item applies to your research, read the appropriate section before selecting a response.

Materials & experimental systems

n/a	Involved in the study	
☐	☒	Antibodies
☐	☒	Eukaryotic cell lines
☒	☐	Palaeontology
☐	☒	Animals and other organisms
☒	☐	Human research participants
☒	☐	Clinical data

Methods

n/a	Involved in the study	
☐	☒	ChIP-seq
☐	☒	Flow cytometry
☒	☐	MRI-based neuroimaging

Antibodies

Antibodies used	Biolegend: anti-CD8α (clone 53-6.7,catalog#100714) dilution: 1/200; anti-KLRG1 (clone 2F1, catalog# 138412/138416) dilution:1/200; anti-CD4 (clone GK1.5, catalog# 100428) dilution: 1/200; anti-Ly5.1 (clone A20, catalog# 110706/110716) dilution:1/200; anti-BrdU (clone Bu20a, catalog# 339808) dilution: 1/100; anti-Ki67 (clone 16A8, catalog# 652411) dilution: 1/100; anti-Ly5.2 (clone 104, catalog# 109822) dilution: 1/200;anti-Annexin V (catalog# 640918) dilution:1/100; anti-IL-2 (clone JES6-5H4, catalog# 503808) dilution:1/200; anti-CD3 (clone 145-2C11, catalog# 100312) dilution:1/200<br>BD Bioscience: anti-CD62L (clone MEL-14, catalog# 553151) dilution: 1/200; anti-CD44 (clone 1M7, catalog# 563114) dilution: 1/200; anti-IFN-g (clone XMG1.2, catalog# 557998) dilution: 1/200; anti-TNF (clone MP6-XT22, catalog# 554420) dilution: 1/100; anti-CD8α (clone 53-6.7, catalog# 551162) dilution:1/200; anti-Vbeta13 TCR (clone MR12-3, catalog# 561542) dilution:1/100.<br>eBioscience: anti-CD25 (clone PC61.5, catalog# 25-0251-82) dilution: 1/200; anti-Thy1.1 (clone HIS51, catalog# 48-0900-82) dilution: 1/200; anti-pAkt1 (Ser473) (clone SDRNR, catalog# 48-9715-42) dilution: 1/100.<br>Cell Signaling: anti-pAkt (clone 193H12, catalog# 4058S) dilution: 1/1000; anti-Ship1 (clone D1163, catalog# 2728S) dilution: 1/1000; anti-Ezh2 (clone D2C9, catalog# 5246S) dilution: 1/1000; anti-Suz12 (clone D39F6, catalog# 3737S) dilution: 1/1000;HRP-anti–mouse IgG (polyclonal, catalog# 7076S) dilution: 1/3000; HRP-anti–rabbit IgG (polyclonal, catalog# 7074S) dilution: 1/3000.<br>Abcam: anti-Jarid2 (polyclonal, catalog# Ab48137) dilution: 1/1000; anti-H3 (polyclonal,catalog# Ab1791) dilution: 1/10000; anti-IgG (polyclonal,catalog# Ab46540) dilution: 1/3000.<br>Millipore: anti-H3K27me3 (polyclonal,catalog# Mab07-449) dilution:1/1000; anti-Gapdh (clone 6C5, catalog# Mab374) dilution: 1/3000.<br>Invitrogen: anti-V5 (clone R960-25, catalog# R960-25) dilution: 1/1000.<br>Santa Cruz Biotechnology: anti-Actin (clone C4, catalog# SC-47778) dilution: 1/1000.
Validation	All antibodies are commercially available and validated by previous studies performed in other and our laboratory.

Eukaryotic cell lines

Policy information about cell lines

Cell line source(s)	B16F10 melanoma was obtained from ATCC, Platinum-E cell line was obtained from Cell Biolabs, B16-hgp100 was obtained from Hanada K. (NCI), NIH.
Authentication	B16-F10 cells and B16-hgp100 were validated by morphology, pigmentation and recognition by gp100-specific TCR transgenic CD8+ T cells.<br><br>B16-hgp100 were distinguished from B16-F10 by blasticidin-resistance.<br><br>Platinum-E cell line was validated by the vendor, and by the authors' assessment of cell morphology and ability to produce retro-viral particles.
Mycoplasma contamination	Platinum-E cells, B16-F10, and B16-hgp100 were validated as being mycoplasma free via a PCR-based assay
Commonly misidentified lines (See ICLAC register)	No commonly misidentified lines were used

Animals and other organisms

Policy information about studies involving animals; ARRIVE guidelines recommended for reporting animal research

Laboratory animals	C57BL/6NCr and B6-Ly5.1/Cr mice were from Charles River; Pmel-1 (B6.Cg-Thy1a/Cy Tg (TcraTcrb)8Rest/J), B6.Cg-Mir155tm1.1Rsky/J (bic/miR-155-) mice and CAS9 (B6J.129(Cg)-Gt(ROSA)26Sortm1.1(CAG-cas9*,-EGFP) mice were from the Jackson Laboratory; Pmel-1 Ezh2fl/fl Cd4cre mice were provided by Zhang Y. from Temple University. Jarid2fl/fl mice were provided by Muljo S. from NIAID, NIH and crossed with Pmel-1 transgenic animals. Phf19–/– mice were obtained from MBP, UC Davis and crossed with Pmel-1 transgenic animals. Sex and age (6-10 weeks) matched mice were employed unless otherwise indicated.
Wild animals	No wild animals were used in the study.
Field-collected samples	No field-collected samples were employed in this study.
Ethics oversight	All mouse experiments were done with the approval of the National Cancer Institute Animal Care and Use Committee.

Note that full information on the approval of the study protocol must also be provided in the manuscript.

ChIP-seq

Data deposition

☒ Confirm that both raw and final processed data have been deposited in a public database such as GEO.

☒ Confirm that you have deposited or provided access to graph files (e.g. BED files) for the called peaks.

Data access links<br>*May remain private before publication.*	https://www.ncbi.nlm.nih.gov/geo/query/acc.cgi) under accession number GSE99918.
Files in database submission	miR155-H3K27me3; miR155-input; Control-H3K27me3; Control-input
Genome browser session (e.g. UCSC)	*Provide a link to an anonymized genome browser session for "Initial submission" and "Revised version" documents only, to enable peer review. Write "no longer applicable" for "Final submission" documents.*

Methodology

Replicates	None
Sequencing depth	The average total read of the 4 chip-seq samples (i.e., miR155-H3K27me3, miR155-input, Control-H3K27me3, and Control-input) is 40.5X10^6 and 99.0% of them can be mapped to the reference mouse genome mm9. Of them, about 95.6% are unique.
Antibodies	anti-H3K27me3 (Cat# Mab07-449) from Millipore.
Peak calling parameters	Peaks were called using the program sicer (version 1.1) with the command-line setting "mm9 1 200 150 0.74 600 .05" (i.e., the window size, gap size, and FDR were set to 200 bp, 600 bp, and 5%, respectively).
Data quality	We filtered the alignments and discarded those alignments with low mapping quality (q-score <30). And we picked the stringent criteria (FDR<0.05) to call peaks.
Software	Basecall was conducted by the Illumina Casava (version 1.7) software, and then reads were mapped to the mouse reference

Software	genome mm9 using bowtie2 (version 2.2.8). Alignments with low mapping quality (<30) were further filtered by samtools (version 1.2), and then were sorted and marked duplication using Picard (version 1.139). Finally, peaks were called using the program sicer (version 1.1) with the command-line setting "mm9 1 200 150 0.74 600 .05".

Flow Cytometry

Plots

Confirm that:

☒ The axis labels state the marker and fluorochrome used (e.g. CD4-FITC).

☒ The axis scales are clearly visible. Include numbers along axes only for bottom left plot of group (a 'group' is an analysis of identical markers).

☒ All plots are contour plots with outliers or pseudocolor plots.

☒ A numerical value for number of cells or percentage (with statistics) is provided.

Methodology

Sample preparation	Mice spleen and thymus were homogenized using 40uM strainer and syringe. Tumors were homogenized using a gentleMACS™ Dissociator and processed with Lympholyte-M solution for lymphocyte enrichment. Naive or whole CD8+ T cells were enriched using enrichment kits from Stem Cell technology or Miltenyi.
Instrument	BD FACS Aria II, BD FACS LSRII, BD FACS Fortessa
Software	BD FACSDivaTM 8.0 was used to acquire the data, Flowjo_v9.9.4 and v10.5 were used to analyze the data
Cell population abundance	Purity after cell sort was determined by Flow Cytometry. The target population purity was higher than 90% for all experiments.
Gating strategy	Cell populations were gated using the following strategy:FSC/SSC–>singlets (FSC-A/FSC-H)–>Live cells–> CD8/reporter or congenic marker. For sorting of non-skewed T cell populations for RNA-seq cells were further selected using a KLRG1-CD62L- gate.

☒ Tick this box to confirm that a figure exemplifying the gating strategy is provided in the Supplementary Information.
