# Supplementary material for: miR-155 harnesses Phf19 to potentiate cancer immunotherapy through epigenetic reprogramming of CD8+ T cell fate
Source: Nat Commun. 2019 May 14;10:2157. doi: 10.1038/s41467-019-09882-8 (PMC6517388; doi:10.1038/s41467-019-09882-8)
Supplement: Supplementary file 4 — Description of Additional Supplementary Files [file 41467_2019_9882_MOESM4_ESM.pdf]

Description of Additional Supplementary Files

File Name: Supplementary Data 1
Description: Top 15 gene datasets negatively enriched in miR-155 cells.

File Name: Supplementary Data 2
Description: Differentially expressed genes by RNA-seq of Pmel-1 transduced with miR-155.

File Name: Supplementary Data 3
Description: Genes uniquely marked by H3K27me3 in control or miR-155 cells.

File Name: Supplementary Data 4
Description: Gene datasets shared between Phf19$^{-/-}$ (negatively) and miR-155 cells (positively).

File Name: Supplementary Data 5
Description: Gene datasets shared between Phf19$^{-/-}$ (positively) and miR-155 cells (negatively).
